# First Insights into Barriers and Facilitators from the Perspective of Persons with Multiple Sclerosis: A Multiple Case Study

**DOI:** 10.3390/ijerph191710733

**Published:** 2022-08-29

**Authors:** Joelle Ott, Nikola Biller-Andorno, Andrea Glässel

**Affiliations:** 1 Institute of Biomedical Ethics and Medical History, University Zurich, Winterthurerstrasse 30, CH-8006 Zurich, Switzerland; 2 Institute of Public Health (IPH), Department of Health Sciences Katharina-Sulzer-Platz 9, Zurich University of Applied Studies (ZHAW), CH-8401 Winterthur, Switzerland

**Keywords:** multiple sclerosis, patient perspective, qualitative research methods, thematic analysis, ICF, DIPEx, patient experience, ethics, narration, multiple case study, source analysis

## Abstract

Multiple Sclerosis (MS) is a complex, lifelong disease. Its effects span across different areas of life and vary strongly. In Switzerland, there is an intense discussion on how to optimize quality of care and patient safety. Patients should be more involved in the management of health care to improve the quality of care from the patient’s perspective and form a more comprehensive perspective. This multiple-case study explores the question of how persons with MS experience and describe functioning related barriers, facilitating factors, and ethically relevant conflicts. To address this from a comprehensive perspective, the MS core set of the International Classification for Functioning, Disability, and Health (ICF) is used as theoretical framework. To explore barriers, facilitators, and relevant ethical issues, different narrative sources were used for thematic analysis and ICF coding: (a) MS transcripts from DIPEx interviews and (b) an autobiographical book of persons living with MS. Insights that were meaningful for daily practice and education were identified: (a) understanding the importance of environmental circumstances based on narrative sources; (b) understanding the importance of a person’s individual life situation, and the ability to switch perspectives in the medical field; (c) respect for PwMS’ individuality in health care settings; (d) creating meaningful relationships for disease management and treatment, as well as building trust.

## 1. Introduction

### 1.1. Multiple Sclerosis

Multiple Sclerosis (MS) is one of the most common organic diseases of the central nervous system. Worldwide, 2.5 million people have been diagnosed with MS. Almost 70% of persons with MS are women [1]. In 2020, nearly 15,000 persons with MS (PwMS) lived in Switzerland [2]. This amounts to 180 affected people per 100.000 of the population. The disease burden of MS was previously estimated for Switzerland in the context of a larger, international consortia. More detailed, subgroup-specific burden estimates are lacking. This knowledge gap is regrettable from a public health perspective. More detailed findings reflecting the disease–severity distribution and age structure of the population of persons with MS (PwMS) are of high relevance for health policy and care providers [3].

The first symptoms develop between the ages of 20 and 40, but MS can also develop in children and people over 40, although this is less common [4]. Depending on the form, women can be up to three times as likely to be affected as men. Prevalence rises as one goes north from the equator.

MS is characterized by demyelination caused by local inflammation. This leads to a decrease in or loss of function of the affected nerve cells. MS is a chronic disease that represents an irreversible presence of disease conditions or damage to the nervous system. The location of these lesions influences the symptoms. The symptoms can be motor, visual, psychological, cognitive, sensibility disorders or many other neurological symptoms. There is a large variation between patients and disease course [4]. Since the progression of the disease, as well as the presentation of symptoms, is very case-specific, the impact of the disease on daily life varies from person to person [5]. One important aspect of how strongly people are affected is how much their mobility is limited and their autonomy is restricted. Ambulation has been self-declared to be the most important body function that is impacted by PwMS. An estimated 75% of PwMS have walking disturbances. This can limit their participation in activities. Environmental factors and personal factors can lighten or exacerbate the impact of these limitations. Numerous devices can aid impaired mobility. These range from canes to motorized scooters to braces that assist ambulation. There are also numerous devices to assist in activities of daily living. These can make it possible for PwMS to retain their independence [5].

A diagnosis of MS fundamentally changes one’s life and often requires a reorganization of many aspects of life [6]. Topics range from rethinking mobility to dealing with personal anxiety as to what the future might hold and do not leave much out in between. To ensure that those affected can gain or retain the best possible quality of life and patient security, a person-centered point of view, a comprehensive bio-psycho-social perspective on health and health care, is needed [7]. In Switzerland, there is an intense ongoing discussion regarding how quality of care and patient safety can be optimized and guaranteed in the future. A “National Report on Quality and Patient Safety in the Swiss Health care System”, commissioned by the Federal Office of Public Health concludes, among other things, that patients should be more involved in their health care management and that the quality of treatment and care should be assessed and improved from the patient’s perspective and from a more comprehensive perspective of health care [8].

### 1.2. International Classification of Functioning, Disability and Health (ICF)

**Figure 1:**
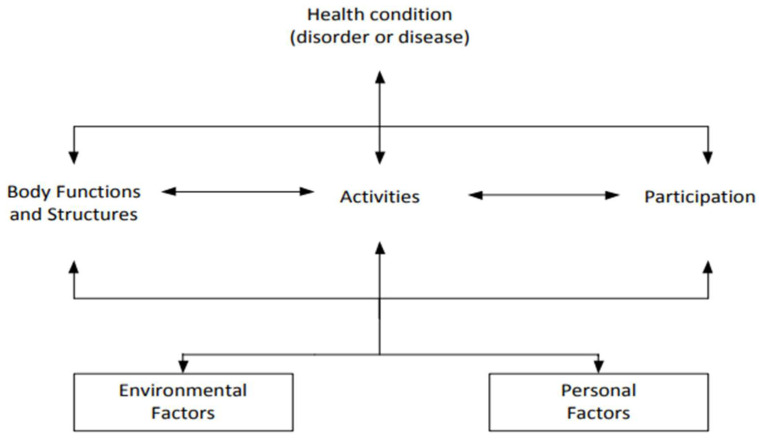
Bio-psycho-social model: This is an open access article distributed under the Creative Commons Attribution License, which permits unrestricted use, distribution, and reproduction in any medium, provided the original work is properly cited [7].

Based on the bio-psycho-social model of health, which stipulates that a health condition is not an isolated entity but needs to be seen in its psycho-social context, the World Health Organization (WHO) developed the International Classification of Functioning, Disability and Health (ICF) (see Figure 1) [7]. This framework and its predecessor focus on functional management and individual chronic disease experiences by considering the whole life situation of a person and aims to provide a standardized language for this purpose. The ICF is based on a model of interaction that provides a system of classification for long-term, non-fatal effects of diseases. Human functioning, or a decrease in human functioning, is portrayed as an interaction between health conditions and contextual factors, which include environmental and personal factors. The different categories of the ICF are related and influence each other in different ways. They can even influence health conditions.

The ICF is organized into two parts: (a) functioning and disability and (b) contextual factors. Contextual factors are divided into environmental factors and personal factors. These components are divided into domains. These domains are further subdivided. Each term is defined starting at the level of the domain and has an alphanumerical code. The environmental factors, which are the focus of this paper, have five domains:

- e1 Products and Technology;
- e2 Natural Environment and Human-Made Changes to Environment;
- e3 Support and Relationships;
- e4 Attitudes;
- e5 Services, Systems and Policies.

These can be qualified as facilitators or barriers depending on whether they support or hinder the person. The same domain or code occurring further down the tree can be a facilitator to one person and a barrier to another, depending on the person’s needs and availability. In this study, the qualification of barrier or facilitator is made based on the PwMS’s description.

This means an environmental factor such as medication (e1101 Drugs) can either be a barrier or facilitator, depending on the availability, impact, and its side-effects. Barriers are defined by the ICF as: “factors in a person’s environment that, through their absence or presence, limit function or create disability. These include aspects such as a physical environment that is inaccessible, lack of relevant assistive technology, and negative attitudes of people towards disability, as well as services, systems and policies that are either nonexistent or that hinder the involvement of all people with a health condition in all areas of life” [7] (p. 222).

Facilitators are described as: “factors in a person’s environment that, through their absence or presence, improve functioning and reduce disability. These include aspects such as a physical environment that is accessible, the availability of relevant assistive technology, and positive attitudes of people towards disability, as well as services, systems and policies that aim to increase the involvement of all people with a health condition in all areas of life. Absence of a factor can also be facilitating, for example the absence of stigma or negative attitudes. Facilitators can prevent an impairment or activity limitation from becoming a participation restriction, since the actual performance of an action is enhanced, despite the person’s problem with capacity” [7] (p. 222).

Personal factors are defined as: “the particular background of an individual’s life and living, and compromise features of the individual that are not part of a health condition or health states” [7] (p. 17), which are so far not classified.

Since the ICF is very extensive, so-called core sets have been developed for different health conditions and health-related situations [7,9,10,11,12]. Condition-specific ICF Core sets for MS were developed by Coenen et al. 2011 [11]. This standard allows for a structured description of persons with specific health conditions, using widely accepted terminology, without the need to navigate the whole ICF [11]. For clinical practice, a case example for PwMS on how to apply the ICF core sets for MS in long-term care, including the context-related factors and the interprofessional treatment team involved, is already available in Switzerland [12]. This paper uses the environmental factors of the comprehensive core set for MS as basis to evaluate barriers and facilitators from two different narrative sources of data: source (1) transcripts of two semi-structured Database of Individual Patients‘ Experiences (DIPEx) interviews, and source (2) a literary autobiographical representation of a person’s life with MS.

### 1.3. Ethical Considerations and Aims of This Case Study

The ICF is a comprehensive theoretical framework for describing and categorizing function, disability, and health, but does not provide a detailed structure for ethical considerations. To strengthen these considerations, this paper uses the four ethical principles of Beauchamp and Childress’s [13], which are widely established and applied in the medical field and health care sector: (a) respect for autonomy, (b) nonmaleficence, (c) beneficence and (d) justice. These principals are not a manual on how to solve a medical ethical dilemma, but a framework that can help to structure a decision-making process. When grappling with an ethical dilemma, it can help to determine which underlying principles are conflicting [14].

The aim of this multiple-case study, designed as a qualitative analysis of narrative sources, is to assess barriers, facilitators, and ethically relevant aspects from the perspective of PwMS, using the common language of the ICF and the MS-specific core set to derive important aspects in PwMS health care. The general research question of this paper is:

“How do PwMS experience and describe barriers, facilitators and ethically relevant conflicts?”

Our research question will be specified as follows:

(a) Which causes are identified, what could possible solutions to functioning-related barriers look like, and which ethical aspects become recognizable?
(b) What consequences and benefits can be found for the collaborative practice in the care of PwMS?

## 2. Materials and Methods

### 2.1. Study Design and Methodology

Our research question is based on a multiple-case study design, as described by Yin 2003 [15], to explore differences within and between cases and enhance data credibility. To make comparisons, the cases were carefully selected so that similar outcomes or contrasting outcomes could be predicted based on theory. In our study, the biopsychosocial model of the ICF was used. We examined three different cases to understand the similarities and differences between the cases. Yin (2003) describes how using multiple case studies either, “(a) predicts similar results (a literal replication) or (b) predicts contrasting results but for predictable reasons (a theoretical replication)” [15] (p. 47).

This work is based on the approach to meanings and understanding focusing on the lived experiences of individuals within a social and personal world. Thus, we follow the interpretive phenomenological analysis (IPA) approach of Smith and Osborn, 2003 [16,17]. IPA is appropriate for discovering how individuals “perceive certain situations they face as they perceive them” [18] (p.429). IPA goes back to Husserl (1913/1983) and his phenomenological position [19].

Therefore, in this multiple-case study, we focused on mixed methods to analyze contextual factors of the ICF. This analysis does not include the whole spectrum of functioning and disability. Additionally, our research questions will be answered using qualitative research methods based on a thematic analysis by Braun and Clarke [20,21] of two different narrative sources:

(1) Two narrative interviews: *Interview A* and *Interview B.*
(2) A literary autobiographical representation: *Book C*.

*Interviews A and B* are semi-structured narrative interviews, conducted for the MS module of the Swiss DIPEx project (www.DIPEx.ch (accessed on 21 August 2022)). The persons being interviewed were informed that the primary use of the interviews was for a website portraying MS, but that they might also be used anonymously for research.

DIPEx is an association of researchers conducting qualitative research into people’s personal experiences of health and illness with a common methodology for conducting semi-structured interviews [22,23]. This narrative method was developed by the Health Experiences Research Group at the Nuffield Department of Primary Care at the University of Oxford. The international DIPEx network comprised thirteen countries implementing their own national DIPEx platforms, based on qualitative studies. The aim of DIPEx is to present a wide spectrum of diverse perspectives on different diseases and health conditions to the public (patients, family caregivers, health professionals, and students) [22,23]. To explain how we use the terms health, disease and illness in this study, we follow the definition of Rovesti et al.: “in the English language, there are three terms to indicate a pathological state: illness, which identifies the personal emotional state connected to the loss of health; disease, which refers to the objective, biological and measurable di-mension of it-strictly linked to the physician’s activity-and sickness, which refers in-stead to the public dimension of the disease and highlights the link between illness and society” [24] (p. 163).

**Figure 2:**
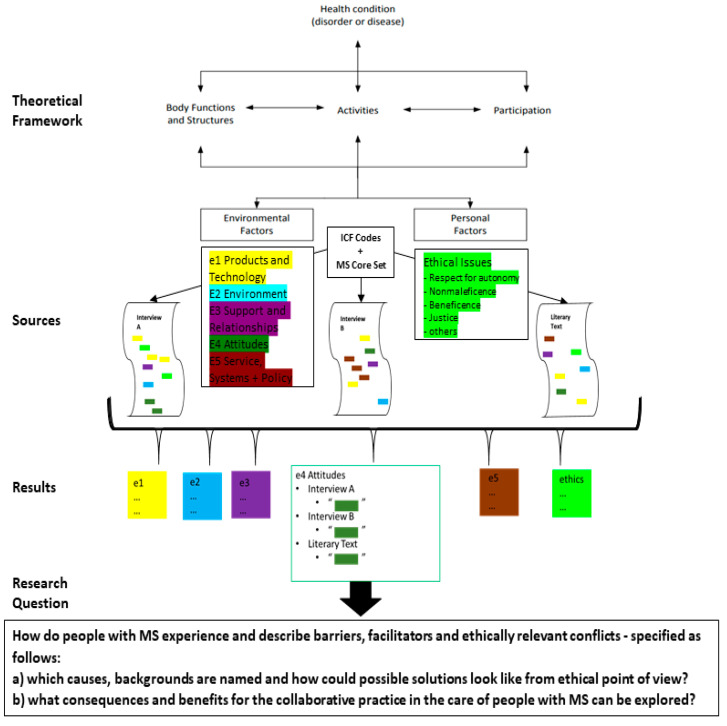
Flowchart and overview of paper’s methods and their integration into the theoretical framework of the bio-psycho-social model of disease and the ICF.

*Book C* is an autobiographical text published in 2019, which describes the author’s journey from Switzerland to Venice in a wheelchair and his inner journey of learning to accept his need for a wheelchair. He writes about his individual experiences living with MS and tells stories about how his wheelchair makes his life easier and more complicated at the same time [25] (see Figure 2).

This multiple-case study was conducted at the University of Zurich in Switzerland from September 2019 to July 2021, and structured in the following steps: selection of cases from the 30 interviews conducted by this point; reading of the book; introduction to the theoretical framework of the ICF and the ICF core sets for MS; training in ICF coding, especially focusing on contextual factors; introduction to the biomedical principles according to Beauchamp and Childress; qualitative research based on thematic analysis according to Braun and Clarke [20,21] to explore the narrative content; structuring and writing the manuscript.

Using a combination of inductive and deductive coding, the barriers, facilitators and ethical aspects mentioned were extracted. The deductive coding derived codes from the comprehensive MS core set developed by Coenen, et al. [11]. As inductive coding, non-core set codes were added where needed.

The ICF is anchored in the bio-psycho-social model for understanding diseases and their effects in daily life situations and their interactions. Additionally, ethical issues, which are part of the personal factors in the ICF, were coded using Beauchamp and Childress’s [13] principles. The results were sorted by ICF domain and used to answer the research question (see Supplementary Materials—as a video presentation).

### 2.2. Role of Researchers

The first author (JO) is a medical student. Her background is focused on more the biological aspect of a disease and less on experiences of illness and the psychosocial evaluation thereof. Her learning has mainly concentrated on objective data and facts. These are an important basis for medical decision-making, and are what medical students are tested on throughout their studies. However, the doctor–patient relationship is also thought to have a significant influence on the treatment outcome. She (JO) is convinced that the doctor’s understanding of a patient’s situation will improve the relationship. This is her motivation for this project. She has never met any of the participants in the DIPEx study, she has never been involved in the treatment of a patient with MS, nor does she have any personal experience with the disease: not as a patient herself, not as friend, and not as a family member of a person diagnosed with MS. The interviewer of these DIPEx interviews is the last author (AG). She is leading the MS DIPEx module with a research background in public health, rehabilitation sciences, and applied ethics. In addition, she has years of experience in a neurorehabilitation setting as a physiotherapist. She is co-supervising this master project. She was not involved in the treatment of the individuals. NBA co-designed the larger study (the DIPEx Module) of which the presented sample is a subset.

### 2.3. Ethics

In response to ethics applications with BASEC-Nr. Req-2018-00050, the Ethics Commissions in Switzerland issued a declaration of non-jurisdiction. This paper is part of the project, that, in accordance with this decision, does not need sanctioning by an ethics commission. The persons interviewed agreed to have their testimonials used for research purposes.

### 2.4. Source Criticism

The narrative sources are first-person accounts, which gives us unfiltered access to the PwMS’s accounts. The semi-structured form of the interviews enhances the comparability, while still leaving room for individual accounts. The literary text does not have predetermined topics, and thus leaves more room for the author to set priorities. All narrative sources are written in German and strongly colored by the narrators and their motivations for creating these sources. The interviews each lasted for about 2 h. The literary text covers 123 pages.

Source 1: Transcripts of two narrative semi-structured DIPEx interviews. The person in *Interview A* is asked how MS has influenced her and her personality; she answers: “completely” and that this is difficult for her. She references barriers more often than facilitators. Her motivation for participation seems to be to show the barriers and to give MS a face, so that one does not only think of a disease, but of the persons affected by the disease.

The person in *Interview B* is more focused on the positive. She gives examples of ignoring inconveniences. This gives the impression that, while she might not leave out the hardships, she might underrepresent their influence. I think that her motivation for participating in this interview was to show that life with MS is still worth living. She says she has good quality of life.

Source 2: A literary autobiographical representation of a person’s life with MS (Book). The author of *Book C* wrote this text to help himself reflect on his experiences with starting to need a wheelchair. While he focuses on tangible barriers and facilitators, he also writes about joy and hardships. This is presented in a rather matter-of-fact manner of writing, which still manages to convey emotions. His main motivation seems to be to foster understanding. Unlike interviews, all his words are deliberately chosen, reread, and then decided upon. This makes for easier reading and clearer lines of thought than an interview.

### 2.5. Data Analysis

The secondary analysis of both narrative sources (DIPEx interviews and book) is based on the thematic analysis by Braun and Clarke [20,21], which is conducted in six phases. The basic idea of the inductive coding is that the procedures of summarizing content analysis are used to develop categories from the material in a step-by-step process. With the inductive approach, the categories are not created before the material is inspected but are derived directly from the material without referring to the theoretical concepts used beforehand. The content analysis in this work aims to explore the barriers, facilitators and ethical aspects that are mentioned. A combination of inductive and deductive approach was chosen to anchor the findings in previous research and to obtain a framework for comparison.

For the deductive part of the coding, the codes were derived from the comprehensive MS core set developed by Coenen, et al. [11] (hereafter referred to as the MS core set). Since the subject was barriers and facilitators, only the ICF codes for environmental factors were used, because the other domains cannot be qualified as facilitators or barriers. Ethically relevant situations were also coded. These MS core-set-derived codes were created as the subject was referenced, as were ICF codes not from the core set when subjects of discussion fell into these categories. All the coding was carried out using the MAXQDA software [26].

### 2.6. Quality Assurance Methods

For quality assurance purposes, communicative validation was used for each step of the process, from developing the research question to the interpretation of the results. The first steps in coding were completed after a training (JO&AG) and peer-reviewing process, coding the content and processing it to ICF categories. The interviews were conducted according to the validated DIPEx methodology [23]. All statements made regarding the content of the narrative sources are directly tied to excerpts from the sources.

## 3. Results

Both narrative sources (*Interview A and B and Book C*) were coded with all five ICF domains of environmental factors and the environmental categories included from the ICF core set. Additional ICF categories from the environmental factors of the whole ICF were added when necessary. The ICF was anchored in the bio-psycho-social model for understanding diseases and their effects in daily life situations and their interactions. Additionally, ethical issues, which are part of the personal factors included in the ICF, were coded using Beauchamp and Childress’s principles [13]. The results were ordered by ICF domain and used to answer the research question. There are a total 314 references throughout the narrative sources, which were translated into ICF language to provide a common basis for comparison.

### 3.1. Overview of Identified Barriers and Facilitators of Environmental Factors

Overall, more facilitators (184) were referenced than barriers (129). The ICF categories e1 Products and Technology (115) and e120 Support and Relationships (120) each received quite a few more references than the e2 Natural Environment and Human-Made Changes to Environment (12) or e4 Attitudes (9), the ICF domains with the fewest references. *Interview A* was the only source which referenced more barriers than facilitators, with almost 60% barriers. Only 17% of *Interview B*’s codes were barriers. In *Book C*, just over a third of all references were barriers.

**Figure 3:**
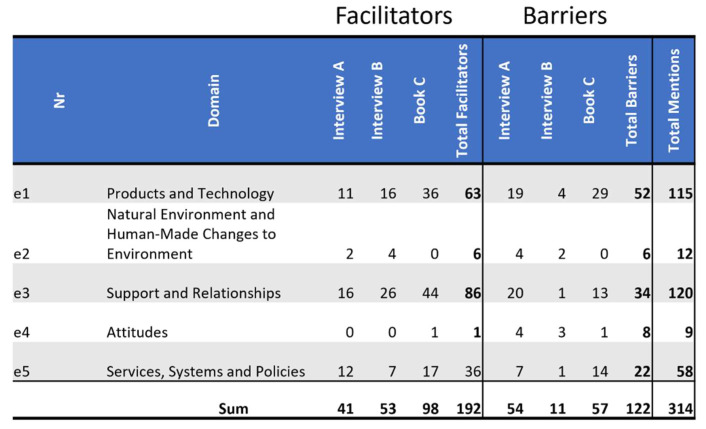
Distribution of codes by ICF domains: This figure shows the frequency of references by domains as facilitators and as barriers. Additionally, it tallies all references as facilitators or as barriers by source, as well as facilitators and barriers both separately and together by domain.

The distribution of code by narrative sources and between barriers and facilitators is shown in Figure 3.

### 3.2. Barriers and Facilitators Identified in ICF Domain e1: Products and Technology

Domain *e1: Products and Technology* has a significant impact on the quality of life of PwMS. This domain is defined by the ICF and includes: “natural or human-made products or systems of products, equipment and technology in an individual’s immediate environment that are gathered, created, produced or manufactured.” The ISO 9999 classification of technical aids defines these as “any product, instrument, equipment or technical system used by a disabled person, especially produced or generally available, preventing, compensating, monitoring, relieving or neutralizing” disability. Any product or technology can be assistive. (See ISO 9999: Technical aids for disabled persons-Classification (second version); ISO/TC 173/SC 2; ISO/DIS 9999 (rev.).) For the purposes of this classification of environmental factors, however, assistive products and technology are defined more narrowly as “any product, instrument, equipment, or technology adapted or specially designed for improving the functioning of a disabled person” [7] (p. 173).

**Figure 4:**
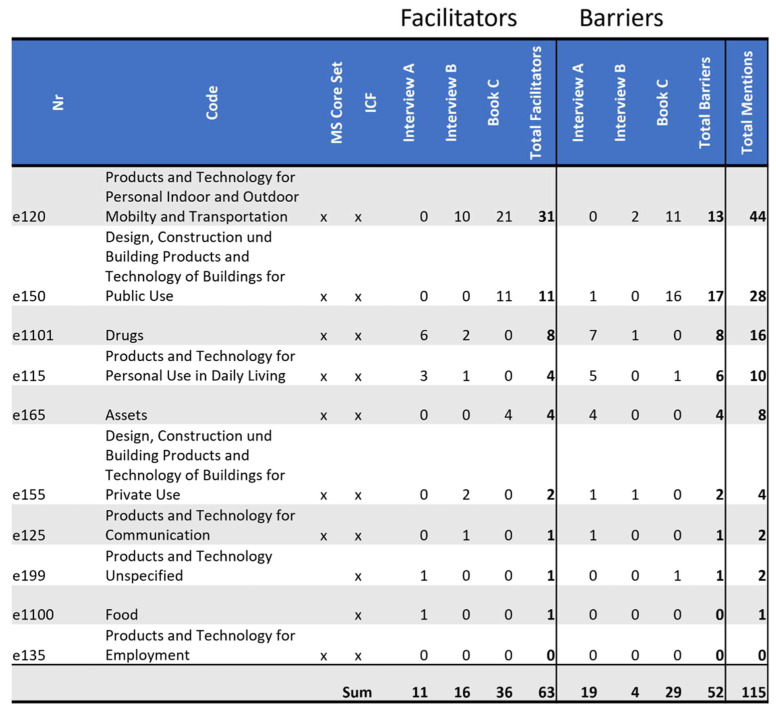
Distribution of codes in the domain *e1 Products and Technology*: This figure shows the frequency of MS core set codes and non-core set codes of this domain, referenced in each source as a facilitator and as a barrier. Additionally, it tallies all references throughout the domain as facilitators or as barriers by source, as well as tallying facilitators and barriers separately and together by code.

This ICF domain *e1: Products and Technology* indicates, as shown in Figure 4, that the same environmental factor, such as the wheelchair, can be both a barrier and a facilitator depending on the perspective and time-related context. An important facilitator that both *Interview B* and *Book C* mention is the wheelchair. They both struggled to accept that they needed one. *Interview B* does not go into detail as to why that was the case for her, but she refused to use one for long after it would have been helpful. When she did start to use it, she saw the difference it made to her quality of life. *Book C* and his daughter saw the wheelchair as a symbol of sickness and dependence. When trying one out for the first time, though, he describes as a sense of freedom. He writes:

*“A feeling of freedom overcame me. I moved effortlessly. I had completely forgotten what freedom of movement felt like. So far, I had only been able to move forward extremely slowly and clumsily. But now? In comparison to before it was like flying.” (Book C)*

*Interview B* attributes some of her quality of life to the wheelchair. Many other large and small tools, such as maps of Venice that showed wheelchair accessibility, a comic that explains MS to children and many more, made the lives of all three persons easier. While the wheelchair is a facilitator for the participants from *Interview B* and *Book C*, *Interview A* does not need one. Most of the tools are only mentioned in one of the sources. Not all tools that are meant to help are helpful, such as the shopping cart for persons in a wheelchair mentioned by *Book C*. This shopping cart did not work for the person in *Book C’s* circumstances. He was shopping with his daughter, for whom the shopping cart was very heavy, especially in addition to him, in his wheelchair, who she also had to push. Additionally, the shopping kept pulling to the side.

The design of rooms in the rehabilitation center that the person in *Interview A* attended had a considerable influence on her. Having her own room was very facilitating for her. 

Having to share her room on later visits was not ideal for her. As with the barriers the person in *Book C* describes, this barrier is not insurmountable but makes life harder for persons who are not the standard user. There are many ways for persons not to be the standard user. The person in *Interview A* most likely had a stronger desire for privacy and profited less from being around other persons than the standard user that the architects had in mind. Person *Book C* was often not the standard user, because of his wheelchair. They benefited from or were hampered by things that are inconsequential to other people, such as having a private room in a rehabilitation facility or the slope of a ramp to the train tracks being slightly too steep to push oneself up.

The person in *Interview A* laments not having adequate vocabulary to describe her fatigue. In German, she feels that there is only one word for tired, while she feels several types of tiredness. What she experiences with her fatigue is different from when other people have a strenuous day and is not always the same for her either. Not having different words for these different experiences is a barrier. It makes it harder for her to communicate what is going on to other people. This makes it harder for these people to understand her situation. She says:

*“Sometimes I sleep and am just as tired when I wake up. Fatigue is like that. It is a malfunction and not a usual tiredness. There are different types of tiredness, totally paralyzing, heavy weighing, every movement is hard work, you know? As if you had hung weights, so ehm, against resistance. Or tiredness that is maybe more in your head, kind of a “prrr fog”, but otherwise one is actually, physically not as much, but up here (the head) is just not useable. Ehm, there are such big differences, are there not?” (Interview A)*

Facilitators do not need to be related a diagnosis or consequences of a disease, such as when *Interview A* talks about food being a thing that brings her happiness and moments of joy. Additionally, sometimes, tools can have unwelcome effects, such as the sanitary napkins that the person in *Interview A* uses for her urinary incontinence. She says that these products influence her vanity and her femininity.

### 3.3. Barriers and Facilitators Identified in ICF Domain e2: Natural Environment and Human-Made Changes to Environment

The domain *e2: Natural Environment and Human-Made Changes to Environment* discusses “animate and inanimate elements of the natural or physical environment, and components of that environment that have been modified by people, as well as characteristics of human populations within that environment” [7] (p. 182).

**Figure 5:**
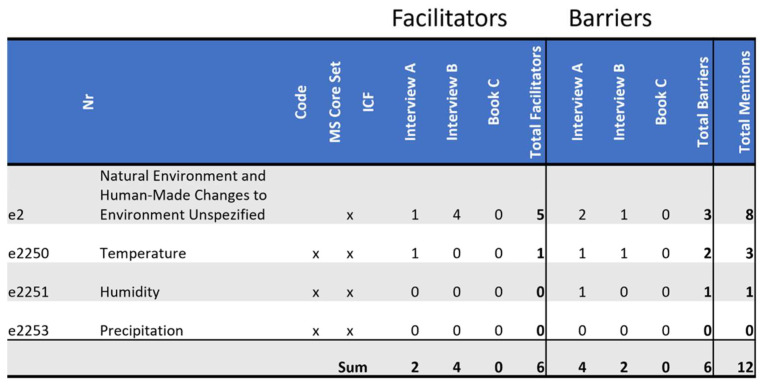
Distribution of codes in the domain *e2 Natural Environment and Human-Made Changes to Environment*: This figure shows the frequency of MS core set codes and non-core set codes of this domain referenced in each source as a facilitator and as a barrier. Additionally, it tallies all references throughout the domain as facilitators or as barriers by source, as well as tallying facilitators and barriers separately and together by code.

Temperature was shown to be an influence in both *Interview A* and *Interview B* (please see Figure 5) For *Interview A* it is mainly heat that she has trouble with. She describes it as feeling *like having hot water pored over oneself.*

*Interview A*’s intolerance for heat and humidity make summers hard, especially because others like to spend time outside during this time of year. Being out of sync this way is a barrier to nurturing friendships. For *Interview B* the cold is the bigger issue.

Both women find being in nature helpful. *Interview B* finds it reenergizing, saying:

*“Then I go to a pond, sit down, stay for a quarter of an hour and there I notice, when I’ve been out in the fresh air. It isn’t like that inside, but water has a calming effect. I recuperate quickly by the ponds.” (Interview B)*

*Interview A* associates it with happiness. The same facilitator brings different benefits to different persons, and this facilitator does not seem to have a direct connection to the disease that these women both have.

### 3.4. Barriers and Facilitators Identified in ICF Domain e3: Support and Relationships

The stories portray *e3: Support and Relationships* as facilitators almost twice as often as barriers (78:42). Per definition this domain is “about people or animals that provide practical physical or emotional support, nurturing, protection, assistance, and relationships to other persons, in their home, place of work, school or at play or in other aspects of their daily activities. The chapter does not encompass the attitudes of the person or people that are providing the support. The environmental factor being described is not the person or animal, but the amount of physical and emotional support the person or animal provides” [7] (p. 187).

**Figure 6:**
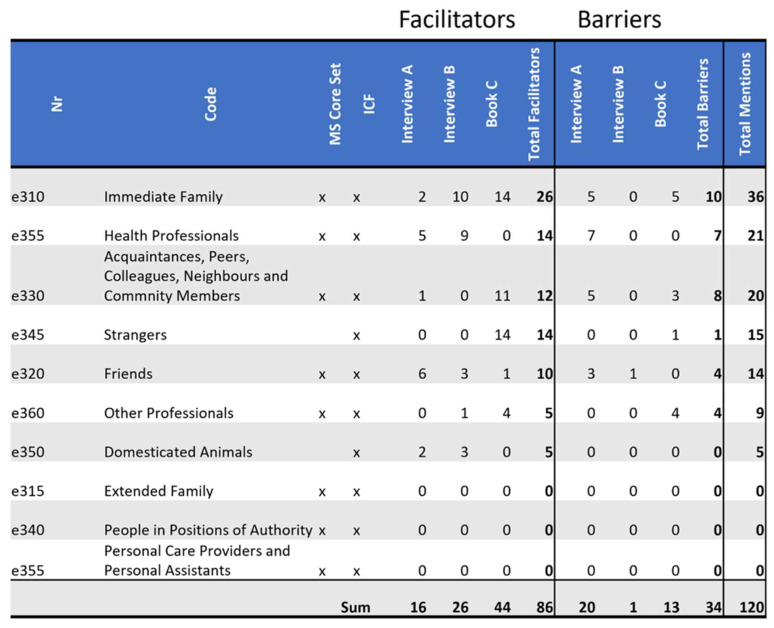
Distribution of codes in the domain *e3 Support and Relationships*: This figure shows the frequency of MS core set codes and non-core set codes of this domain referenced in each source as a facilitator and as a barrier. Additionally, it tallies all references throughout the domain as facilitators or as barriers by source, as well as tallying facilitators and barriers separately and together by code.

The domain *e3 Support and Relationships* is referenced the most as a facilitator (please see Figure 6). In *Interview A*, it is also the domain that is mentioned the most as a barrier. The example of *Interview A’s* relationship shows that a diagnosis such as MS, with the changes it brings, can strain a relationship. In this example the relationship possibly became a barrier, because it did not survive the changes. 

*Interview A’s* conversation about husbands leaving wives after such a diagnosis goes to show that she was aware that the continuation of her marriage was not guaranteed.

*Book C’s* relationship with his children plays a central role in his book. He writes about two of his children:

*“Joy and Charley <…> helped significantly, that I do not see the wheelchair as a dis-aster anymore, but as a tool, that is sometimes needed.” (Book C)*

All also mentioned persons outside of their family. For *Interview A*, these are mainly friends. These are facilitators in 6 out of 9 references. For *Interview B*, her neurologist played an important facilitating role in her journey with MS. All references to him are as a facilitator. *Book C* talks about strangers and work colleagues. The strangers were facilitators 14 out of 15 times. These strangers told him about routes that were more wheelchair accessible, and strangers helped him get up after he fell. Colleagues were facilitators in 11 out of 14 references. He writes about them jumping up to offer him help and making sure he is seen when moving through a crowd.

For *Interview A*, many relationships with doctors were barriers because they did not take her needs into account, apart from her direct medical needs. These stories give the impression of her being reduced to her diagnosis. This is in stark contrast to the person in *Interview B’s* relationship with her neurologist, where she feels that he knows what she wants and what she needs. She describes them deciding whether she needs to take cortisone as follows:

*“Sometimes he looks at me and he knows that I don’t really like cortisone. But when he says there is nothing else, we need to do it, then I know, he isn’t just doing this, he’s prescribing it for a certain reason. <…> he says: “I know you don’t like it, but…” Then I say, well, then we’re doing it.” (Interview B)*

*Book C* presented two quite different experiences in two different stores that sold wheelchairs. The main differences seemed to be customer friendliness and competence. Additionally one of the salespeople talked about AHV (old age and survivors’ insurance) wheelchairs. The person in *Book C* really did not like this. How things are named has an impact on people. The person in *Book C* is not old enough to receive AHV, but needs a wheelchair. The salesperson in the store where the author of *Book C* had the unpleasant experience seemed to have a standard user in mind, who was different from the one standing in front of him. Still he did not change his approach. He also did not seem to consider or ask about the needs of this customer.

Being reduced to someone who needs a wheelchair is shown as a barrier more than once in *Book C*. On the other hand, people being considerate that he is in a wheelchair, for example sitting down to be on eye level, was described as a facilitator. The statement, shows that people take his wheelchair into account:

*“Surprisingly there was always someone there, that helped me with my wheelchair. Usually people offered help, without me asking.” (Book C)*

Both *Interview A and B* described pets that are facilitators. Their being facilitators does not directly have anything to do with their MS. They enjoy the animals’ company, like many people without MS.

*Interview A*, *Interview B* and *Book C* all put great emphasis on the people around them. The 120 references to this domain show this. People can be great facilitators, but unfortunately also barriers.

### 3.5. Barrieres and Facilitators Identified in ICF Domain e4: Attitudes

The domain *e4: Attitudes* includes the following aspects and is defined by the ICF as follows: “attitudes that are the observable consequences of customs, practices, ideologies, values, norms, factual beliefs and religious beliefs. These attitudes influence individual behavior and social life at all levels, from interpersonal relationships and community associations to political, economic, and legal structures; for example, individual or societal attitudes about a person’s trustworthiness and value as a human being that may motivate positive, honorific practices or negative and discriminatory practices (e.g., stigmatizing, stereotyping, and marginalizing or neglect of the person). The attitudes classified are those of people external to the person whose situation is being described. They are not those of the person themselves. The individual attitudes are categorized according to the kinds of relationships listed in Environmental Factors Chapter 3. Values and beliefs are not coded separately from the attitudes as they are assumed to be the driving forces behind the attitudes” [7] (p. 190).

The woman in *Interview A* describes one specific attitude that has been a large barrier for her: society’s focus on work as a central element of who we are. This leads people to ask strangers what they do for a living to get to know them. This can quickly lead to persons who do not work feeling othered, which, in person *Interview A*’s example, led to her isolating herself from strangers, so she did not have to explain why she does not work to strangers during small talk. She says:

*“Yes, after years of avoiding new contacts, among other things simply because I. Yes, just like that, always MS, everywhere MS. Somehow the first question when you get to know someone new is always, what do (you) do in our culture. And then you are always there very quickly, you don’t work, and you don’t see my MS anymore when you look at me, so what is the problem? Well, then I am already explaining again.” (Interview A)*

Person *Book C* gives numerous examples of people seeing him in his wheelchair and offering help, from clearing a path for him in a crowd to bringing him food from a buffet. While this description of society’s attitude towards persons who visibly have special needs is a facilitator for him, it could also become a barrier for persons whose needs are not as obviously visible.

The distribution of codes quite clearly shows that attitudes were referenced as barriers a lot more than as facilitators (8/9). This could be interpreted as attitudes tending to be barriers more often than facilitators. It is also possible, and maybe even more likely, that attitudes that hinder are easier to name than those that help.

**Figure 7:**
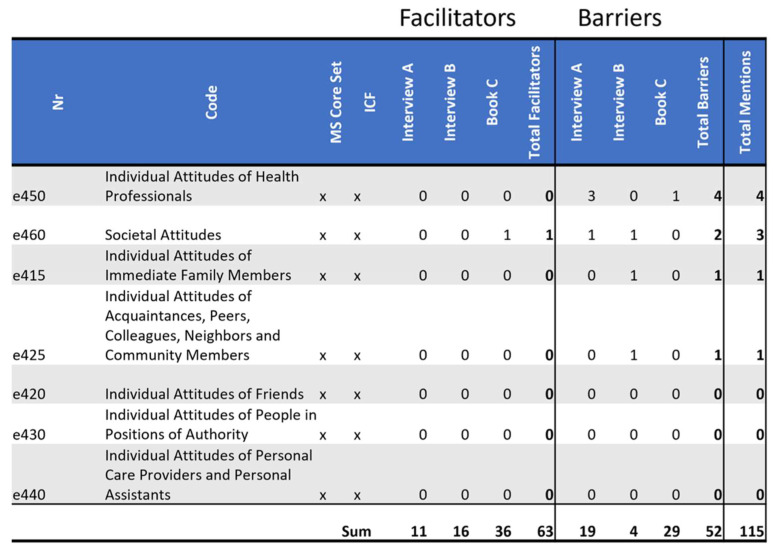
Distribution of codes in the domain *e4 Attitudes*: This figure shows the frequency of MS core set codes and non-core set codes of this domain referenced in each source as a facilitator and as a barrier. Additionally, it tallies all references throughout the domain as facilitators or as barriers by source, as well as talling facilitators and barriers separately and together by code.

No codes outside of the MS core set were used. A total of 4/7 of the MS core set codes were referenced; please see Figure 7.

### 3.6. Barrieres and Facilitators Identified in ICF Domain e5: Services, Systems and Policies

This section leads into domain *e5: Services, Systems and Policies,* the contents of which are described as follows: “1. Services that provide benefits, structured programs and operations, in various sectors of society, designed to meet the needs of individuals. (Included in services are the people who provide them). Services may be public, private, or voluntary, and may be established at a local, community, regional, state, provincial, national, or international level by individuals, associations, organizations, agencies, or governments. The goods provided by these services may be general or adapted and especially designed. 2. Systems that are administrative control and organizational mechanisms, and are established by governments at the local, regional, national, and international levels, or by other recognized authorities. These systems are designed to organize, control, and monitor services that provide benefits, structured programs, and operations in various sectors of society. 3. Policies constituted by rules, regulations, conventions, and standards established by governments at the local, regional, national, and international levels, or by other recognized authorities. Policies govern and regulate the systems that organize, control, and monitor services, structured programs, and operations in various sectors of society [7] (p.192)”.

*Book C* shows many examples of public transportation being adapted to his needs sitting in a wheelchair. Having a hotline to organize assistance for getting into specific trains is one of the many cases where these adaptions were facilitators. When it was unclear where his reserved seat was, because of a not-documented seat change due to his wheelchair and other such examples, imperfections in the adaptations to his needs were barriers. In this example he switched train cars twice with children, luggage, and a wheelchair. For the person in *Interview B*, public transportation where she lives is not good enough for her to solely rely on it.

Nobody described *e580 Health Services, Systems and Policies* as not being functional. *Interview A* and *Interview B* have different experiences with the degree of personalization. *Interview B* discusses the rehabilitation center she attended at the time of the interview:

*“Here it’s good in any case. If something were not to work, or is too strenuous, then I can say so. I think, up here everyone can say so, and then you do something else.” (Interview B)*

All three narrative sources (A, B, C) report receiving financial support from different forms of insurance, without claiming it to beinsufficient.

**Figure 8:**
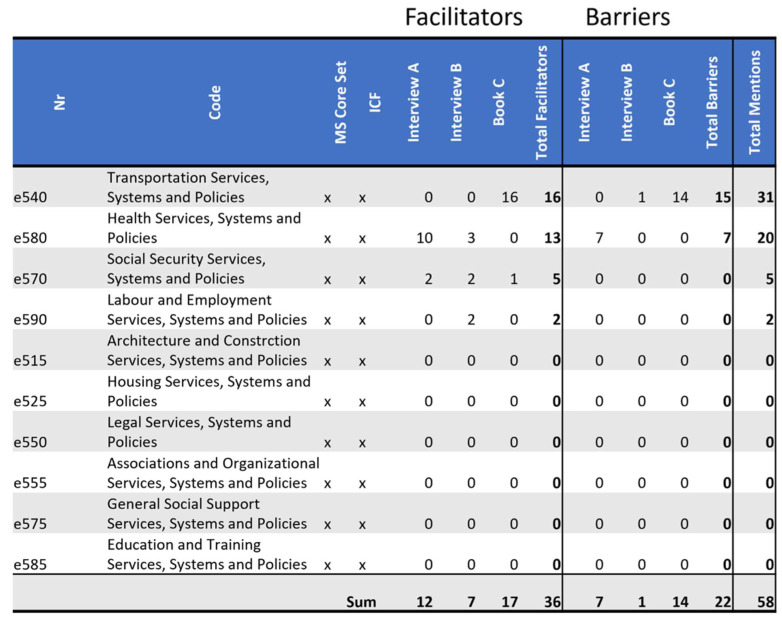
Distribution of codes in the *domain e5 Services, Systems and Policies*: This figure shows the frequency of MS core set codes and non-core set codes of this domain referenced in each source as a facilitator and as a barrier. Additionally, it tallies all references throughout the domain as facilitators or as barriers by source, as well as tallying facilitators and barriers separately and together by code.

Most codes of the MS core set in this domain were not referenced (*e550 Legal Services, Systems and Policies e515, e525 Housing Services, Systems and Policies, e575 General Social Support Services and Policies, e585 Education and Training Services, Systems and Policies*); please see Figure 8.

### 3.7. Identified Ethical Issues

Unlike the other topics, this chapter does not follow the ICF, since the ICF does not explicitly take ethical issues into account. Instead this chapter discusses ethical issues as conflicts between Beauchamp and Childress’s four ethical principles, [13] which are widely established and applied in the bio-medical field and health care sector:

1. Respect for autonomy.
2. Nonmaleficence.
3. Beneficence.
4. Justice.

This chapter indicates the situations described in the interviews and the literary text, where not all of Beauchamp and Childress’s ethical principles were acted upon.

Only *Interview A* describes a lack of beneficence. In two examples, situations made her uncomfortable and kept her from obtaining the optimum amount out of either setting, even though both were meant to be for her benefit. In the third example, the neurologist did not make a diagnosis, because he did not find it medically relevant. He did not see the benefits that it could have had for her psychologically.

In both *Interviews A* and *B*, situations are described where *e355 Health Professionals* do not fulfill the principle of autonomy. In one example, they do not allow the PwMS to set her own goals. In another, they override the PwMS’s preferences. In *Book C*, the *e540 Transportations Services, Systems and Policies* tried to take away the author’s autonomy by telling him where to spend his layover.

Both instances mentioned persons not adhering to the principle of nonmaleficence: e355 Health Professionals said inopportune things. When the person in *Interview B* asked whether she had MS, she was told it could also be a brain tumor. The person in *Interview A* was told:

*“Ach, if you did not always cry, you would be a pretty woman…” (Interview A)*

## 4. Discussion

With this multiple-case study, we explored the following question: “How do persons with MS experience and describe barriers, facilitators and ethically relevant conflicts?” We looked at the backgrounds that are discernible in this by means of narrative source analysis of experiences of PwMS, and the consequences for health care management for persons with MS. Based on the translation of these narrative sources into the common language of the systematic bio-psycho-social structure of the ICF, it was possible to identify patient-health-care-relevant context factors as barriers and facilitating factors and to compare them to the MS ICF core sets as a practical tool for a comprehensive health care management. There are several aspects that we would like to discuss here.

### 4.1. Quantitative Distribution

For most comparisons between the number of barriers and facilitators referenced per code, there were more facilitators (13:9). The categories where barriers outweighed facilitators tended to have fewer references than those where facilitators outweighed barriers. There was an average of almost 18 references per code where facilitators outweighed barriers and an average of almost 8 where barriers outweighed facilitators.

### 4.2. Ethical Issues

Based on the systematic coding of the narrative sources with the ICF, ethically relevant aspects of the PwMS became clear, which can generally be assigned to the contextual factors. A detailed description of this is not found in the ICF. Based on the four biomedical principles, a classification became possible.

The focus of the ethical issues was *e355 Health Professionals* not respecting autonomy or not fulfilling the potential for beneficence. The principle of justice was not touched upon, perhaps because the focus in all three accounts was very much on the individual. The portrayed circumstances were varied and the underlying causes were not always entirely clear. Chu’s research also emphasizes that ethical thinking and acting are important components of narrative subjectivity, including the ability to understand others and overcome differences, understand and reflect on values, to compromise, to reach a trade-off between different values, and, finally, to undergo an interdisciplinary collaboration with different professions [27].

Still some inferences and suggestions to circumvent such incidents can be made and similar ideas are portrayed in the following chapters.

### 4.3. Lessons Learned Based on an Analysis of Narrative Sources of PwMS

Based on the results of this multiple-case study, the first insights of lessons learned from the Swiss experiences of PwMS regarding barriers and facilitators are postulated in the following sub-chapters. To indicate the Swiss perspective, we connect our results to the findings of Bechtold’s et al., as shown in their study “Quality through patients’ eyes” [28].

One of our goals is to describe the factors we explored, and whether they had a positive influence as a facilitator or a negative influence as a barrier, in collaboration with the interdisciplinary treatment of PwMS, which is addressed where suitable in the following sub-chapters. For this the descriptions in these narrative sources were used to make inferences as to why some aspects were barriers and others were facilitators, and why the same aspects could be a barrier for one person and a facilitator for another person, or in a different circumstance.

#### 4.3.1. Understanding the Importance of Environmental Circumstances Based on Narrative Sources

As shown in the study of Chu et al., narrative thinking is not meant to arrange events in chronological order, but to place the disease/hospitalization in the context of life to construct the temporality of the disease [27]. In Chu’s words: traditional “biomedicine orientation (is) seeking the truth and facts, narrative thinking focuses on the authenticity of patients and diseases, which refers to touching stories and revelations in medical care, including the value of life, the beauty of human nature, selfless dedication, resistance to disease, family emotions, loss, rebirth, etc.” [27]. This aspect is also presented by Chiu et al. They identified barriers to health care, which they divided into three phases of utilization: (a) in the pre-visit phase, the most frequently cited barrier was transportation; (b) in the phase during the visit, the quality of communication was the biggest problem; (c) in the phase after the visit, the failure to refer for follow-up treatment was what the biggest barrier [29].

The importance of understanding individual circumstantial experiences of illness became especially clear when the PwMS telling their stories in these sources spoke of the tools they used and their strategies for managing life with a chronic disease. The better adapted the strategies were to the individual’s circumstances, the more beneficial they were. This ranged from a comic book that was ideal to explain MS to children to wheelchair shopping carts that were too heavy to push in addition to a wheelchair. They also included sanitary napkins designed for menstruation that also work for incontinence, but leave much to be desired.

Understanding circumstances is not only important for providing tangible aid, but also when lending a helping hand. When it is overdone, it can diminish a person’s autonomy and the necessary degree of care turns into over-care or even paternalism. It is always important to consider the individual and his or her subjective experiences and needs, and not to reduce persons with MS to their diagnosis.

Facilitators do not have to be connected to the person’s MS. The person in *Interview A* finds that food brings her joy. *Interview A* and *B* both describe pets as facilitators. Research has shown that pets can have positive effects on psychological as well as physical wellbeing [30]. There is no impression that either of these facilitators have anything to with them having MS or any disabilities resulting from this. This does not make their effect less valuable. Broadening our view of what can help somebody to include things that are not disease- or disability-specific could allow us to think of such things. It could also help the affected people look for facilitators further away from their ailment or find tools that are especially helpful to them even if they are not specifically designed for PwMS, such as the easy-to-clean kitchen mentioned in *Interview B*.

Both *Interview A* and *Book C* describe finding it uncomfortable when they get the impression of being reduced to a PwMS or a person in a wheelchair.

Researchers have investigated different circumstances to try and find solutions. This includes researchers who explored the circumstances of falls in wheelchair users [31], but also topics that are far from from this paper’s topic and focus on very different circumstances, such as a paper that investigated the circumstances around women’s entry into sex work to find ways to help targeted HIV prevention [32,33]. Sometimes it is small things that can have a large impact. Hammel et al. gives examples of simple devices, such as remote controls or pagers, which have a large benefit, because they meet a person’s needs [34].

#### 4.3.2. Understanding the Importance of Person’s Individual Life Situation–Ability to Switch Perspectives in the Medical Field

The sources show multiple examples of persons seeking to understand. This ranges from criticism of medical jargon that is difficult to understand to grandchildren informing themselves about what is going on with their grandmother using comic books, as well as to the person in *Interview A* wanting a diagnosis whether it was relevant to the treatment or not. The sources also show the importance of being understood. The person in *Interview B* feels judged for her unsteady gait. She also feels her doctor knows that she does not like taking cortisone. She describes him prefacing his recommendation that she take cortisone with: “I know you don’t like it, but…” This makes her more receptive when he does suggest she take it. This reciprocal understanding makes for a good working relationship between patient and doctor.

While a PwMS will undoubtedly play a large role in their treatment, one needs to make sure not to reduce the person or the treatment to the diagnosis. As much as a there is a person sitting in the wheelchair in person *Book C’s* story, not just a purse, there is more to each PwMS than their diagnosis. This line of thinking can lead to other ways to help and increase understanding for the person. In the Swiss context Berchtold et al. were able to show, in their study on health care quality in Switzerland, that “professionally perceived quality on the one hand and patient experience in spoken and written narratives on the other hand represent two fundamentally different perspectives: It is precisely this difference in perspective that patients address in the narrative interviews” [28] (p. 13). As Berchtold et al. show that no one doubts the basic competence of the physicians. Not even the young woman who had to wait seven years for her diagnosis. However, all of them react with unease when they do not find their questions and concerns taken up, when they feel pressed into medical categories and when they do not feel perceived as individuals [ibid].

Furthermore, as explored by Schmid et al., the patient’s perspective plays a critical role in health care and quality assurance. During an episode of a disease, patients come into contact with many therapists involved in inpatient and/or outpatient care. They can review the entire chain of care. As a therapist one must learn to understand the patient’s point of view and put oneself in his or her shoes. PwMS often try to do their best or to improve or maintain their quality of life by themselves. Patients develop coping skills; therefore, healthcare professionals mentioned coping strategies for everyday actions [35].

#### 4.3.3. Respect for PwMS’ Individuality in Health Care Settings

Not everyone is alike. Just because someone fits into a category, this does not stop them from being individual. For example people presumed that the person in *Interview A* needed company, because others had. Wheelchair accessibility did not mean the same thing for the author *Book C* as it did for the people declaring whether trains were wheelchair accessible or not. While 75% of PwMS have impaired mobility, this also means that 25% do not. As with this symptom, the whole clinical presentation differs from patient to patient [32]. In Fakolade et al.’s research over 70% of participants with MS cited lack of choice and control of physical activity and level of engagement as a barrier to participation in physical activity programs [36]. This is just one example for a possibility to increase the impact and accessibility of valuable programs by increasing their adaptability to individual needs. For example, Kayes et al. [37] looked at what helped or hindered physical activity in PwMS. While they also found disease-related variables that had an influence, they found barriers that had no direct link to the disease. They propose that health professionals gaining an understanding of these individual barriers could improve the PwMS’ physical activity and barriers to accessibility. The literature review of Chiu et al. recommend that clinicians find individualized solutions with PwMS who face transportation barriers to care, including reimbursements for travel and home medication delivery [29]. In other words seeing the PwMS as a person with attributes not related to the disease allows for health professionals to be more effective, since their patient might have barriers that are not related to the disease.

From a more general and Swiss health care perspective, the findings of Berchtold et al. show that one important, issue when building trust and increasing the understanding of good health care, is offering sufficient space, attention, and appreciation to the individual patient’s situation [28]. They show that patients find it very unpleasant to be perceived only as an object instead of as a “whole person” and to be pressed into medical categories as quickly as possible. This is important in the acute setting, but especially in the aftercare setting, where the patient’s needs are manifold. In principle Berchtold et al. recommend that medical and nursing professionals are more systematically trained to deal with differences in perspective than is currently the case in Switzerland [ibid]. Specifically in relation to MS, Mayo et al. (2021) substantiate Bechtold’s claims and emphasize that PwMS and their health care professionals can benefit from a structured and comprehensive MS-specific education. Education can address the process of addressing unmet health care needs in health care settings and ultimately lead to a higher quality of life for people with MS [38].

#### 4.3.4. Relationships Are Meaningful for the Disease Management and Treatment to Build Trust

All three narrative sources of PwMS, that were studied in this paper, provide multiple examples of facilitating relationships and indicate the meaning and relevance of these relationships for PwMS. These range from *Interview B’s* neurologist to *Book C’s* children. *e3 Support and Relationships* is the domain with the most references and the domain that most strongly favors facilitators. The hardships that arise in a relationship when one partner is diagnosed with MS and how these relationships can be helpful has been researched previously [39,40,41]. As shown by Fakolade et al., when PwMS and their caregivers ranked what they most needed to participate in physical activities, programs that included the family caregivers ranked first for family caregivers and second for PwMS, showing the central role that relationships play [36]. Kassie et al. found that social support or lack thereof was mentioned in almost all the interviews, demonstrating the importance given to this topic. Those who had support viewed it as indispensable to the management of their disease [42]. For PwMS, relationships are an important factor in managing their disease.

The importance of relationships has also been pointed out by Haubrick et al., who explored the lived experience of adults with MS. It became clear that while medical professionals and family members do not always offer support, respondents indicated that, in contrast, they find help and friendship in MS support groups. One woman stated, “I’ve become an outcast to most of the people I knew before.” Participants unanimously valued social engagement when coping with illness [43].

Obtaining insight into significant factors of PwMS also requires good medical relations, according to Chu. This includes communication between doctors and patients, as well as across medical professions. The exchange of life experiences and dialogue between doctors and patients is one of the steps in the promotion of good medical care [27].

### 4.4. Potential Benefits from a Methodological Point of View

What do the insights based on this multiple-case study from a methodological and learning theory perspective indicate for medical students? They offer various benefits due to their systematic, structured approach using different narrative sources: (1) *interview (A and B*) and (2) a literary autobiographical representation by a *book (C*). These insights can sharpen reflections regarding:

(a) The contextual reference, starting from a diagnosis-related focus and demonstrated by the real-life situations of the personal accounts of PwMS in the DIPEx interviews.
(b) The intensity of the in-depth content analysis of ICF’s (interaction model and classification for functioning and disability) real-life descriptions on a bio-psycho-social basis.
(c) The systematic consideration of different factors favoring or aggravating the life situation from the perspective of persons affected by MS and its influence on health care.
(d) A potential comparability of statements based on a common language, known for the clinical picture (ICF) in rehabilitation and standards developed for this purpose to describe functioning and disability (ICF core set for MS) as a reference.
(e) The inclusion of biomedical principles as an analysis, aiding in the recognition of ethically relevant aspects, that are woven into real-life situations.

### 4.5. Limitations

This multiple case-study offers some initial insights into the barriers and facilitators for PwMS and does not allow us to generalize, but can be assumed to provide a basis for initial hypotheses. The methodology aims to show areas of interest without claiming to assess their transferability to other situations. This includes this paper’s focus on PwMS. This qualitative approach, based on a case study design and without saturation of data, does not allow for generalization, but it provides a basis for developing initial hypotheses.

Our three narrative sources focusing on barriers and facilitators do not reference all the topics of functioning and disability in the MS core set. This does not provide a complete view of the PwMS’s situations. The payoff is that this methodology is more open to topics that would not have come up if the MS ICF core set had been used as a questionnaire. Another limitation for the scope of the statements for Switzerland, with its three national languages, German, French and Italian, is that the three sources (A–C) were German-speaking and do not allow for transference to the other two language regions.

All three narrative sources are from a PwMS’s perspective. To obtain an objective picture of a situation, one needs multiple points of view. Obtaining such an objective picture was never the objective of this paper. MS is also called the disease with a thousand faces for a reason. The goal was to explore the PwMS’s perspectives based on their subjective experiences regarding barriers and facilitators, which are ethical issues for those living with MS, to receive an initial overview.

## 5. Conclusions

Barriers and facilitators impact the success of health care and are, therefore, significant in medicine. This paper, whose method is strongly influenced by the humanities, while still being a medical master’s thesis, should be seen as a supplement to the more deficit-oriented way that medicine is often practiced. It is not a criticism of this way of looking at disease, but an additional perspective. In addition to the more standard interventions, such as medication, it is important to be aware of environmental factors and their impact on a PwMS’s life. Putting all this together is a challenge that medical professionals face every day. This paper lists four lessons that are meaningful for the daily practice and education of medical and health professional students:

- Understanding and importance of environmental circumstances based on narrative sources.
- Understanding and importance of a person’s individual life situation and ability to switch perspectives in the medical field.
- Respect for PwMS’ individuality in a health care setting and not reducing persons to their diagnosis.
- Constructing meaningful relationships and building trust for the disease management and treatment.

These are not entirely novel, but are important to reiterate and could use additional research, especially to reconsider these aspects’ inclusion in medical and health care education programs and learning plans, as suggested by Chu [27]. With our insights, we join Berchtold [28] and invite medical professionals to switch their perspective of the medical field to that of a person being treated instead of the person providing the treatment. The factors explored here can be supported by tools such as DIPEx, which share a wide variety of patients’ perspectives. Important questions include their transferability to other PwMS and possibly to persons with other chronic diseases, as well as the possibility of deepening medical students’ reflections, looking beyond diagnosis towards a bio-psycho-social perspective. Even if many of these obstacles ultimately require the implementation of lasting policy changes, others can be more easily removed by increasing awareness among health care professionals and responding to patients’ needs.

## Data Availability

Not applicable.

## Supplementary Materials

The following supporting information can be downloaded at: https://www.mdpi.com/article/10.3390/ijerph191710733/s1, Video S1: English presentation of study: “Barriers and facilitators from person’s perspective with multiple sclerosis—A case-based source analysis” at European Congress of NeuroRehabilitation (ECNR) 2021 jointly with 27. Jahrestagung der Deutschen Gesellschaft für Neurorehabilitation (DGNR) 08–11 December 2021.
